# Crack Cocaine Smoke Induces Tissue Degeneration in Rat Submandibular Glands by Toll-like Signaling Pathway

**DOI:** 10.3390/pathophysiology32030032

**Published:** 2025-07-02

**Authors:** Lorrany da Silva Avanci, Daniel Vitor de Souza, Gabriel Carvalhal de Aguiar, Thiago Guedes Pinto, Barbara dos Anjos Rosario, Milena de Barros Viana, Yasmin Alaby Martins Ferreira, Viviane Carlin Cordaro, Luciana Pellegrini Pisani, Daniel Araki Ribeiro

**Affiliations:** 1 Department of Biosciences, Institute of Health and Society, Federal University of Sao Paulo UNIFESP, Rua Silva Jardim, 136, Room 332, Vila Mathias, Santos 11050-020, SP, Brazil; avanci.lorrany@unifesp.br (L.d.S.A.); daniel.vitor@unifesp.br (D.V.d.S.); g.aguiar@unifesp.br (G.C.d.A.); guedes.pinto@unifesp.br (T.G.P.); barbara.arosario@gmail.com (B.d.A.R.); mviana@unifesp.br (M.d.B.V.); yasmin.alaby@gmail.com (Y.A.M.F.); pisani@unifesp.br (L.P.P.); 2 Institute of Criminalistics of Sao Paulo, Sao Paulo 01255-000, SP, Brazil; vivivanecarlin@gmail.com

**Keywords:** crack cocaine, salivary glands, submandibular salivary gland, immunoexpression

## Abstract

Background: This study investigated the impact of crack cocaine smoke exposure on the submandibular salivary gland of Wistar rats. Methods: The animals were distributed into four groups: control (CTRL); 25 mg exposure (CK25); 50 mg exposure (CK50); and 100 mg exposure (CK100). The animals were exposed to crack cocaine smoke once a day for five consecutive days. Results: Exposure to crack cocaine smoke-induced histopathological changes in submandibular salivary glands in all groups under exposure. The immunohistochemical analysis demonstrates that exposure to crack cocaine smoke led to an increase in BCL-2 and P16 expression in all groups exposed to crack cocaine (*p* < 0.05). The analysis of Ki-67 expression revealed a significant increase in immunoreactive cells across all exposure groups (*p* < 0.05). Although MYD88 expression was observed in all crack cocaine-exposed groups, only the group treated with the highest dose (100 mg) exhibited a statistically significant increase compared to the control group (*p* < 0.05). Conclusions: In summary, this study demonstrates that exposure to crack cocaine smoke-induced tissue degeneration in the submandibular salivary gland, increasing cellular senescence and promoting compensatory cell proliferation in Wistar rats.

## 1. Introduction

*Erythroxylum coca* is a South American plant species that contains a high concentration of several compounds, including the alkaloid cocaine, which is a highly addictive psychoactive stimulant [1]. After extraction, cocaine can be found in two types of psychoactive drugs: cocaine hydrochloride (cocaine salt) and crack cocaine, the latter being the product of a three-step process (melting, cooling, and solidification) that closely resembles small yellowish-brown or beige stones [2].

Crack consumption had its debut in Brazil in 1991 and, since then, its addiction has increased and become a serious public health and socioeconomic problem [3]. Typically, crack cocaine users belong to vulnerable and marginalized groups that experience social and economic disadvantages. Additionally, crack cocaine consumption could be influenced by other factors such as social, psychological and genetic factors, exposure to violence, sexual abuse, parental drug use and family conflict [4]. Among the deleterious events that may occur due to this drug abuse, health problems stand out as representatives of potential comorbidities, including the development of oral lesions, such as aphthous lesions, gingivitis, angular cheilitis and hyposalivation, among other dental disorders [5].

In this context, when addressing oral health problems, salivary glands are important exocrine organs that impact oral homeostasis, as they produce, modify, and secrete saliva, an oral health key fluid composed of a number of substances [6]. The major salivary glands, the submandibular one is responsible for most of the unstimulated saliva production [7]. As for the salivary secretion process, it is known that saliva is stimulated by parasympathetic stimuli upon myoepithelial cells, which leads to contraction around the acini and consequent fluid secretion into the lumen [8].

Although there are studies that address the correlation between hyposalivation and crack cocaine use, the available literature on the topic remains limited, and the existing research focuses on measuring salivary secretion in individuals who use crack cocaine [9]. A previous investigation established a correlation between hyposalivation and crack cocaine use, that is, a significant reduction in salivary flow rate has been observed among users compared to non-users [10]. However, it is still unclear whether the effects of crack cocaine occur directly in the glandular parenchyma or interfere with the autonomic control of salivary flow. Considering the points raised about the abuse of crack cocaine, its potential effect on salivary flow and the limited amount of studies addressing this topic, the aim of the present study was to assess the crack cocaine smoke exposure effects on the submandibular salivary glands of Wistar rats in order to better elucidate this matter.

## 2. Materials and Methods

### 2.1. Illicit Drug

The crack cocaine used in this research was obtained from seizures by the Civil Police of the State of São Paulo and provided by the Judiciary Police Inspectorate (DIPO) from the Central Criminal Forum of Barra Funda in the city of São Paulo, based on an order from Judge (Proc. N 361/19—DIPO 5.1.1). Gas chromatography associated with mass spectrometry was used to perform a qualitative chemical analysis of the drug. The results showed that the samples contained 64% of cocaine.

### 2.2. Animals and Experimental Design

A total of 40 adult (8-week-old) male Wistar rats weighing approximately 300 g were used in this study. The rats were obtained from the Center for Development of Experimental Models for Medicine and Biology (CEDEME) at the Federal University of São Paulo—UNIFESP. The use of animals followed the criteria of the 3R’s guidelines, and the entire study was approved by the Animal Use Ethics Committee under number 2704130922. The animals were housed in the animal facility at UNIFESP—Campus Baixada Santista, under a 12-h light-dark cycle and maintained at a controlled temperature of 22 ± 1 °C with commercial diet and water ad libitum. The rats were then randomly divided into four groups, as follows: control (CTRL, *n* = 10): Animals that received no treatment; CK25 group (*n* = 10): animals exposed to a dose of 25 mg crack cocaine; CK50 group (*n* = 10): animals exposed to a dose of 50 mg crack cocaine; CK100 group (*n* = 10): animals exposed to a dose of 100 mg crack cocaine.

The illicit drug was administered through crack cocaine smoke inhalation. This method was used to simulate how crack cocaine is typically consumed by its human users. The protocol and equipment used for exposure to crack cocaine smoke were based on the study by Araújo et al. [11]. At the end of the experimental period, the animals were anesthetized with a combination of ketamine (75 mg/kg), xylazine (10 mg/kg), fentanyl (0.5 mg/kg) and acepromazine (1 mg/kg), which were administered intraperitoneally. After induction of deep anesthesia, the submandibular salivary glands were collected and the animals were euthanized through the heart section method.

### 2.3. Histopathological Analysis

The evaluation of the glandular tissue was based on the criteria outlined by Souza et al. [12]. This included an assessment of secretion accumulation, vacuolation, changes in cuboidal epithelial cells of the ducts, the presence of inflammatory infiltrates, hemorrhage, abnormal acinar pigmentation, areas of necrosis and areas of cellular degeneration.

### 2.4. Histomorphometric Analysis

The study adopted the parameters established by De Aguiar and colleagues [13], which were modified to meet the specific requirements of enumerating serous and mucous acini in the submandibular salivary gland. The Axio Observe D1 Zeiss^®^ inverted microscope (Carl Zeiss AG—Oberkochn, Germany), along with the Axio Vision 4.8 software, was used for this purpose. The H.E stained slides were used to count the serous and mucous acini, with *n* = 5 per group, and 10 fields per animal were photographed at 400× magnification.

### 2.5. Immunohistochemistry

The immunoexpressions of Ki-67, P16, and BCL2 proteins were evaluated at this stage. The primary antibodies were diluted as follows: Ki-67 (Biocare Medical^®^ 1:150, Pacheco, CA, USA), p16 (Santa Cruz Biotechnology^®^ 1:50, Dallas, TX, USA), and BCL2 (Santa Cruz Biotechnology^®^ 1:500). The slides were then incubated overnight at 4 °C in a dark chamber. The slides were washed twice with PBS and then incubated with the biotinylated secondary antibody for 30 min using the Starr Trek Universal HRP Detection Kit from Biocare Medical^®^. Optical microscopy was used to conduct analyses of the nuclear protein Ki-67, as well as the cytoplasmic expressions of BCL2 and P16. The cytoplasmic and nuclear expression of these markers in the acini and ducts was calculated by covering 10 microscopic fields per animal at a magnification of 400×.

### 2.6. Western Blot—Toll-like Pathway

Submandibular samples (100 mg) were homogenized in 0.8 mL of extraction buffer containing 100 mM EDTA, 100 mM Tris, 10 mM sodium pyrophosphate, 100 mM sodium fluoride, 10 mM sodium orthovanadate, 2 mM phenylmethylsulfonyl fluoride, and 0.1 mg/mL aprotinin. The samples were then placed on a membrane and incubated with primary antibodies MYD88, p-NFxbp65, Claudin 2, and TRAF-6. The optical density was immediately measured at a wavelength of 450 nm using a microplate reader. A calibration curve was constructed by treating the results with linear regression, and the correlation coefficient (R^2^) and straight-line equation were obtained using Microsoft^®^ Excel 2019 software.

### 2.7. Statistical Analysis

The statistical analysis of the results obtained from the histopathological, histomorphometric, and immunohistochemical techniques (Ki67, P16, and BCL-2) and Western Blotting (P65, MYD8, Claudin2, and TRAF6) was performed using BioEstat software version 5.3 (Instituto Mamirauá, Tefé, AM, Brazil). The one-criterion Anova test was used, followed by Tukey’s post-hoc test to verify the significance of the results, adopting 95% (*p* < 0.05) as a significance parameter. The results were expressed as mean ± standard deviation (SD).

## 3. Results

### 3.1. Histopathology

Histopathological evaluation was performed on the submandibular salivary gland parenchyma of animals in this study. The severity of histopathological changes was classified using the scores presented in Table 1. No significant changes were observed in the submandibular salivary gland of the control group. The parenchyma of the gland showed normal cell morphology.

In the CK25 group, the animals exhibited tissue changes compared to the other groups exposed to crack cocaine and CTRL. Whilst areas of salivary retention were observed in the interstitial space, cellular vacuolization, areas with necrosis and small hemorrhagic foci were observed. Additionally, abnormal morphological modification of the cuboidal cells of the glandular ducts and more pigmented serous acini were seen.

The CK50 and CK100 groups exhibited extensive cellular vacuolation, salivary retention in the interstitial space, hemorrhagic foci and abnormal morphological modification of the cuboidal cells of the glandular ducts. Furthermore, greater pigmentation of the serous acini was observed. However, no areas of necrosis were detected in the glandular parenchyma of these groups.

The crack cocaine exposure groups demonstrated significant statistical differences (*p* < 0.05) when compared to the CTRL group. Additionally, the CK25 group demonstrated a significant difference (*p* < 0.05) when compared to the groups exposed to crack (CK50 and CK100). Nevertheless, no statistically significant differences were observed between the CK50 and CK100 groups. The numerical results can be found in Table 1.

### 3.2. Histomorphometry

Histomorphometry of serous acini showed a decrease in density in the CK25 group compared to the CTRL group (*p* < 0.05). The CK50 group also exhibited a decrease in serous acinar density compared to the CTRL and CK25 groups (*p* < 0.05). Similarly, the CK100 group showed a reduction in serous acinar density only in relation to the CTRL group (*p* < 0.05). The result of the histomorphometric analysis of mucous acini in the CK25 group showed an increase in mucous acini density compared to the CTRL group and all crack cocaine exposure groups (CK50 and CK100) (*p* < 0.05). The group CK50 showed a decrease in mucous acinus density compared to the groups CTRL and CK25 (*p* < 0.05). Similarly, the CK100 group demonstrated a decrease in mucous acini density compared to the CTRL group and the CK25 group (*p* < 0.05). Table 2 provides detailed results regarding serous acini. The results are shown in Table 2.

### 3.3. Immunohistochemistry BCL-2, P16 and Ki67

**Figure 1:**
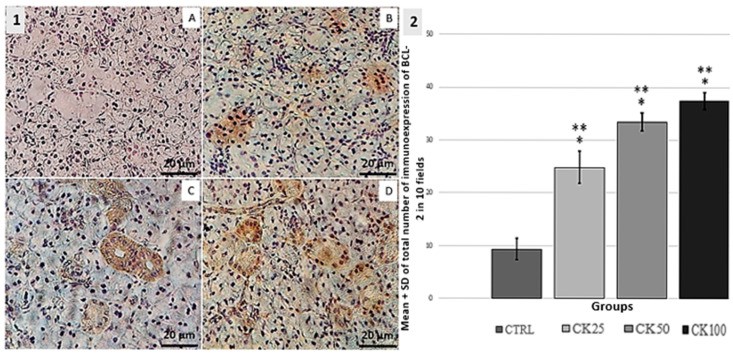
1-(**A**–**D**): Immunohistochemical analysis of BCL-2 expression in the submandibular salivary gland. The control group (CTRL—**A**) was compared to groups exposed to crack cocaine doses of 25 mg (CK25—**B**), 50 mg (CK50—**C**), and 100 mg (CK100—**D**). The CK25 group showed marked serous acini, while the CK50 group showed marked cuboidal epithelial cells and mucous acini. The CK100 group showed marked serous and mucous acini. 2-Graphic: The total number of labeled cells in the submandibular salivary gland was measured by the mean ± standard deviation SD in 10 microscopic fields per slide. In groups CTRL, CK25, CK50 and CK100 (*n* = 5), significant changes were observed at *p* < 0.05 when compared to the CTRL group (*) and among exposure groups (**).

BCL-2. The CK25 group exhibited increased expression of BCL-2 immunoreactive cells compared to the CTRL group. The groups CK50 and CK100 exhibited a higher expression of BCL-2 immunoreactive cells compared to the groups CTRL and CK25. Statistical analysis revealed no significant difference (*p* < 0.05) between the CK50 and CK100 groups. These results can be seen in Figure 1.

**Figure 2:**
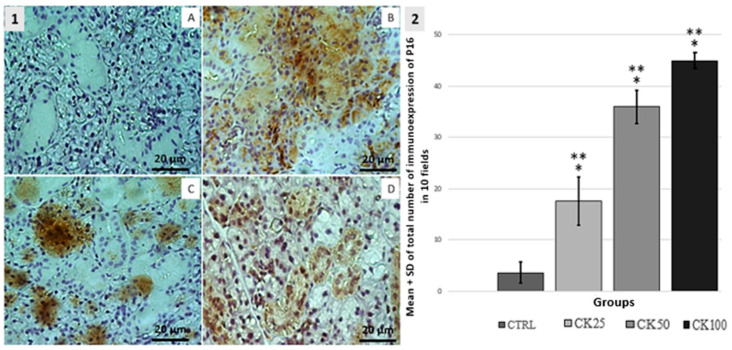
1-Immunohistochemical analysis of P16 expression in the submandibular salivary gland. The results suggest a dose-dependent effect of crack cocaine exposure on P16 expression. The control group (CTRL—**A**) was compared to the groups exposed to crack cocaine doses of 25 mg (CK25—**B**), 50 mg (CK50—**C**), and 100 mg (CK100—**D**). The CK25 group showed marked serous and mucous acini, while the CK50 group showed marked mucous acini and cuboidal epithelial cells. The CK100 group showed marked serous and mucous acini. 2-Graphic: Mean ± standard deviation (SD) of the total number of cells marked in the submandibular salivary gland were calculated for 10 microscopic fields per slide in groups CTRL, CK25, CK50, and CK100 (*n* = 5). Any significant changes at *p* < 0.05 were noted when compared to the CTRL group (*) and among exposure groups (**).

P16. The CK25 group exhibited increased expression of P16-immunoreactive cells. A statistically significant difference was observed when compared to the CTRL, CK50 and CK100 groups (*p* < 0.05). The expression of P16-immunoreactive cells was significantly increased in the CK25, CK50, and CK100 groups compared to the CTRL group (*p* < 0.05). Additionally, the CK100 group exhibited a significant increase in P16 cell expression compared to the CK25 and CK50 groups (*p* < 0.05). The results indicate that all groups exposed to crack cocaine showed an increase in the expression of P16 immunoreactive cells, as shown in Figure 2.

**Figure 3:**
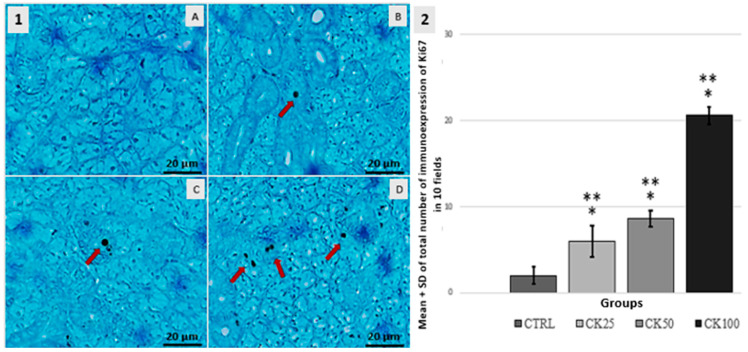
1-Immuno-histochemical analysis of Ki67 expression on the submandibular salivary gland. The cuboidal epithelial cells were marked in all groups, with the addition of mucous acini in the CK100 group. The control group (CTRL—**A**) was compared to groups exposed to crack cocaine at doses of 25 mg (CK25—**B**), 50 mg (CK50—**C**) and 100 mg (CK100—**D**). Immunoreactive cells were indicated by red arrows. 2-Graphic: The mean ± standard deviation (SD) of the total number of labeled cells in the submandibular salivary gland was measured in 10 microscopic fields per slide for groups CTRL, CK25, CK50 and CK100 (*n* = 5). Significant differences were observed at *p* < 0.05 when compared to the CTRL group (*) and among exposure groups (**).

Ki67. In the CTRL group, a low number of Ki67-positive cells were observed. In the CK25 group, there was a statistically significant increase in Ki67 expression in serous and mucous acinar cells, as well as in ductal cuboidal cells (*p* < 0.05). The CK50 group exhibited an increase in the expression of cells that are immunoreactive for Ki67. This expression was observed in serous and mucous acinar cells, as well as ductal cuboidal cells. As for the CK100 group, a significant increase in the expression of immunoreactive cells for Ki67 was observed when compared to the CTRL CK25 and CK50 groups (*p* < 0.05). The results can be seen in Figure 3.

### 3.4. Toll-like Pathway

**Figure 4:**
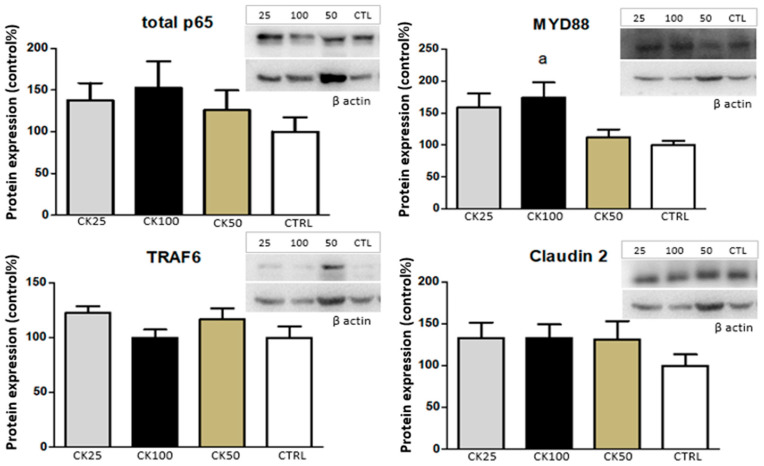
The mean and standard deviation of protein expression levels of pro-inflammatory cytokines were measured in the submandibular salivary gland of four groups (*n* = 5): control (CTRL), and groups exposed to crack cocaine at doses of 25 mg (CK25), 50 mg (CK50), and 100 mg (CK100). Statistical analysis revealed significant differences (*p* < 0.05) between the CTRL group and the crack-exposed groups (a).

The expression of MYD88 was higher in the CK100 group compared to the CTRL one (*p* < 0.05). Although all groups (CTRL, CK25, CK50, and CK100) showed expression of TRAF6, there were no significant differences when groups were compared to each other. Similarly, Claudin 2 expression was observed in all groups (CTRL, CK25, CK50, and CK100) with neither distinction nor statistically significant differences among the groups (*p* > 0.05). Regarding total p65 protein, its expression was detected in all experimental groups, and although CK100 and CK25 exhibited a numerical increase compared to the control, the difference was not statistically significant (*p* > 0.05). These results can be seen in Figure 4.

## 4. Discussion

The use of crack cocaine can have devastating effects on an individual’s health, culminating in several pathological changes [14]. In this sense, it is coherent to state that this illicit drug is a significant public health issue with serious socioeconomic consequences worldwide. Herein, there is still a need for a lot of research in this field so the toxicological mechanisms can be better elucidated.

The histopathological analysis of the control group showed typical characteristics of a normal submandibular salivary glandular tissue. In contrast, the analysis of the groups exposed to crack cocaine revealed significant morphological changes in the glandular parenchyma, demonstrating the cytotoxicity of crack cocaine. Following exposure to crack cocaine, areas of vacuolation were observed in the serous acinar cells, indicating possible cellular degeneration. Additionally, there has been observed an accumulation of secretion in the interstitial space, which culminated in the obstruction of the salivary flow and interfered with the gland function. The observed changes in the glandular parenchyma included greater acinar pigmentation, which may be related to abnormal protein production; changes in the morphology of ductal cells, and may indicate dysfunction in saliva secretion and transport; small hemorrhagic areas, suggesting ruptured blood vessels; and regions of necrosis as a result of cell death. Particularly, it was observed the presence of necrotic areas in the CK25 group, which was associated with the toxicological effects of drugs administered at low concentrations. In certain cases, these concentrations can induce focal necrosis before the tissue is able to manifest compensatory or adaptive responses [15]. Following the rationale, it is reasonable to conclude that the lower dose resulted in necrosis and subsequent cell death in specific regions of the submandibular salivary gland. Taken together, all of the listed changes, including necrosis, are consistent with the cytotoxic effects of crack on the salivary gland. Certainly, this can lead to interference with cellular integrity and salivary gland dysfunction, resulting in xerostomia symptoms [16,17]. Previous studies suggest that crack cocaine can have a direct impact on various types of cells, including oral mucosa, lymphocytes, corneal, brain, liver and kidney cells, potentially leading to the development of various lesions [18,19,20]. The combination of the results of this study with other published works demonstrates the cytotoxic effects of crack cocaine on the submandibular salivary gland, highlighting the harmful potential effects of the drug on oral health.

It has been established that mammalian cells can trigger adaptive responses to preserve function and structure when cellular damage is of low intensity [21]. One of these adaptations is atrophy, which is characterized by a decrease in tissue or organ mass. In this study, the histomorphometric analysis showed a decrease in serous acinar density in all crack cocaine doses (25 mg, 50 mg, and 100 mg) compared to the control group. The decrease in serous acinar density can be attributed to the cytotoxicity of crack cocaine. Particularly, the group exposed to the 25 mg dose exhibited an increase in mucous acini density compared to the control group and the other groups exposed to different doses of crack cocaine. This observation can be explained by the hypothesis that serous acinar cells may be more sensitive to crack cocaine cytotoxicity than mucous acinar cells. This difference in sensitivity may account for the decrease in serous acinar density and the increase in mucous acini density in the group exposed to the 25 mg dose of crack cocaine. Histomorphometric analysis of the groups exposed to 50 mg and 100 mg of crack cocaine revealed atrophy in both serous and mucous acinar cells of the glandular parenchyma. This observation suggests that, at such doses, cells may be going through adaptation due to the cytotoxicity of crack cocaine. Bozorgi and colleagues [22] demonstrated that salivary gland atrophy is a common side effect of chemotherapy and radiotherapy for tumors in the head and neck region. These therapeutic procedures can damage gland cells, leading to their death and a subsequent decrease in saliva production. Sleep deprivation in rats has also been shown to cause atrophy of the mucous acini of the sublingual salivary glands, although the tissue morphology of the mucous acini remained intact [13]. Additionally, a study on crack/cocaine use suggests that changes in cell differentiation may be related to vasoconstriction, which reduces blood flow to tissues [23]. This tissue ischemia may lead to cellular adaptation and, in more severe cases, to necrosis.

To better understand the effects of crack cocaine at the cellular and molecular levels, we were able to investigate some biomarkers closely related to apoptosis, proliferation, inflammation and cellular senescence. The immunohistochemical analysis showed that exposure to crack cocaine at all doses resulted in increased expression of BCL-2 in cells. Considering that BCL-2 is a crucial regulator of apoptosis, it is logical to state that the presence of BCL-2 immunoreactive cells indicates the activation of the apoptosis inhibitory regulation system. Previous studies have demonstrated that cell cycle and DNA repair proteins, such as RAD9, interact with BCL-2 and BCL-xL through the BH3 receptor, promoting apoptosis after DNA damage [24]. Taken as a whole, crack cocaine increased the expression of BCL-2 in groups exposed to the drug, suggesting an antiapoptotic response in submandibular salivary gland cells.

The results of Ki-67 expression analysis in the crack cocaine exposure groups revealed a significant increase compared to the control group, particularly in the highest dose group (100 mg). Cell proliferation exhibited greater expression in the group exposed to a dose of 100 mg of crack cocaine. Farhangkhoee et al. [25] evaluated the expression of Ki-67 in burns of varying degrees and found a correlation between increased thermal injury and greater expression of Ki-67. The authors suggest that analyzing Ki-67 expression could serve as an index to determine the depth of the burn [25]. Previous studies, such as those by De Aguiar et al. [13] and Souza et al. [12], also suggest that this increased expression of Ki-67 may be related to a compensatory mechanism following tissue injury. This observation supports the hypothesis that the administered dose of crack cocaine used in this study was harmful, as it triggered the compensatory mechanism in response to tissue damage. In parallel, in all exposure groups, an increased expression of P16 was observed. P16 is a protein that regulates the cell cycle during the G1 to S transition and is related to cellular senescence, which is characterized by the irreversible arrest of cell growth [26]. Taken together, crack cocaine can increase P16 expression and cellular senescence, which may have various clinical implications, including disease development and accelerated cellular aging.

Finally, MYD88 expression was detected in all experimental groups; however, a statistically significant increase was observed only in the group exposed to the highest dose of crack cocaine (100 mg). This suggests that a higher level of exposure is required to trigger a more pronounced activation of the Toll-like pathway in the submandibular salivary gland under the present experimental conditions. In contrast, Souza and colleagues [12] reported increased expression of some inflammatory markers (MYD88, TRAF6, and p65) in the liver tissue of rats exposed to lower doses of crack cocaine (18 mg and 36 mg). It is important to note that in their study, the illicit drug was administered via the intraperitoneal route, which may have enhanced systemic absorption and contributed to the effects observed at lower doses. These differences highlight the importance of the administration route and exposure level in modulating inflammatory responses to crack cocaine. Further studies are required to determine the precise molecular mechanisms by which crack affects the expression of inflammatory proteins in the submandibular salivary gland.

## 5. Conclusions

In conclusion, our results indicate that exposure to different doses of crack cocaine smoke (25 mg, 50 mg, and 100 mg) can cause morphological changes in the glandular parenchyma of the submandibular salivary gland in rats, as well as dysregulation of the apoptotic and proliferative processes and activation of the Toll-like pathway.

## Figures and Tables

**Table 1:** Histopathological analysis of submandibular salivary glands.

GROUPS	*n*	SCORE				
		0	1	2	3	
CTRL	7	5	2	0	0	
CK25	7	0	0	4	3	*, **
CK50	7	0	0	5	2	*
CK100	7	0	0	5	2	*

* significant change (*p* < 0.05) in comparison with the control group (CTRL); ** significant change (*p* < 0.05) among the groups exposed to crack cocaine at a dose of 25 mg (CK25), 50 mg (CK50) and 100 mg (CK100).

**Table 2:** Mean ± SD and total number of acini per group in submandibular salivary glands of animals exposed to crack cocaine.

Groups	Serous Acini Mean ± SD	Mucous Acini Mean ± SD
CTRL	357.8 ± 12.67	119.6 ± 17.24
CK25	247.8 ± 14.06 *, **	166.6 ± 13.22 *, **
CK50	298.8 ± 25.26 *, **	76.20 ± 24.31 *, **
CK100	274.2 ± 6.3 *	73.2 ± 3.49 *, **

* significant change (*p* < 0.05 compared to the control group (CTRL). ** significant change (*p* < 0.05) intergroup, i.e., when compared to a dose of 25 mg (CK25), 50 mg (CK50) or 100 mg (CK100).

## Data Availability

Data obtained and analyzed in the work are publicly available.

## References

[1] White D.M., Huang J.P., Jara-Muñoz O.A., Madriñá N.S., Ree R.H., Mason-Gamer R.J. (2021). The Origins of Coca: Museum Genomics Reveals Multiple Independent Domestications from Progenitor *Erythroxylum gracilipes*. Syst. Biol..

[2] Zacca J.J., Botelho E.D., Vieira M.L., Almeida F.L., Ferreira L.S., Maldaner A.O. (2014). Brazilian Federal Police drug chemical profiling—The PeQui project. Sci. Justice.

[3] Miguel A.Q.C., Madruga C.S., Simões V., Yamauchi R., da Silva C.J., Abdalla R.R., McDonell M., McPherson S., Roll J.M., Mari J.J. (2018). Crack cocaine users views regarding treatment with contingency management in Brazil. Subst. Abuse Treat. Prev. Policy.

[4] Perrenoud L.O., Oikawa K.F., Williams A.V., Laranjeira R., Fischer B., Strang J., Ribeiro M. (2021). Factors associated with crack-cocaine early initiation: A Brazilian multicenter study. BMC Public Health.

[5] Spezzia S. (2020). Oral manifestations from crack consumption. J. Oral Investig..

[6] Kessler A.T., Bhatt A.A. (2018). Review of the Major and Minor Salivary Glands, Part 1: Anatomy, Infectious, and Inflammatory Processes. J. Clin. Imaging Sci..

[7] Chibly A.M., Aure M.H., Patel V.N., Hoffman M.P. (2022). Salivary gland function, development, and regeneration. Physiol. Rev..

[8] Ozdemir T., Srinivasan P.P., Zakheim D.R., Harrington D.A., Witt R.L., Farach-Carson M.C., Jia X., Pradhan-Bhatt S. (2017). Bottom-up assembly of salivary gland microtissues for assessing myoepithelial cell function. Biomaterials.

[9] Chaiben C.L., Batista T.B.D., Penteado C.A.S., Barbosa M.C.M., Ventura T.M.O., Dionizio A., Rosa E.A.R., Buzalaf M.A.R., Azevedo-Alanis L.R. (2021). Salivary proteome analysis of crack cocaine dependents. Arch. Oral Biol..

[10] Antoniazzi R.P., Sari A.R., Casarin M., Moraes C.M.B., Feldens C.A. (2017). Association between crack cocaine use and reduced salivary flow. Braz. Oral Res..

[11] Araújo M.S., Melo I.S., Silva N.K.G.T., Souza F.M.A., Santos-Neto J.G., Pacheco A.L.D., Cavalcante C.M.B., Freitas-Santos J., Ferreira-Rodrigues A.K.B. (2018). Crack cocaine inhalation induces cardiacatrophy and facilitates limbic-motor seizures in mice submitted to subconvulsive dose of pilocarpine. Int. J. Adv. Sci. Res. Manag..

[12] Souza D., Rosarioa B., Casagrandea B., Viana M., Estadella D., Peres R., Seabra Pereira C.D., Peres R. (2022). Histopathological and inflammatory response in multiple organs of rats exposed to crack. Int. J. Environ. Health Res..

[13] De Aguiar G.C., Souza A.C.F., de Souza D.V., Neto M.M., Le Sueur-Maluf L., de Moraes Malinverni A.C., Antunes H.K.M., Ribeiro D.A. (2023). Degenerative changes induced by paradoxical sleep deprivation in rat sublingual glands. Eur. Arch. Otorhinolaryngol..

[14] De Queiroz Constantino Miguel A., Sandi Madruga C., Simões V., Yamauchi R., da Silva C.J., McDonell M., McPherson S., Roll J., Laranjeira R.R., de Jesus Mari J. (2019). Contingency management is effective in promoting abstinence and retention in treatment among crack cocaine users with a previous history of poor treatment response: A crossover trial. Psicol. Reflex. Crit..

[15] Vidonja Uzelac T., Tatalović N., Mijović M., Miler M., Grahovac T., Oreščanin Dušić Z., Nikolić-Kokić A., Blagojević D. (2024). Ibogaine Induces Cardiotoxic Necrosis in Rats—The Role of Redox Processes. Int. J. Mol. Sci..

[16] De Lima R.C., Ferraz P., Chaiben C.L., Fernandes Â., Grégio A.T., Machado M.N., Azevedo-Alanis L.R., de Lima A.S. (2016). Genotoxic and Cytotoxic Potential of Smoke Crack Cocaine on the Epithelium of the Human Oral Mucosa. J. Dent. Indones..

[17] Guedes Pinto T., Viana M.B., Cury P.R., Martins M.D., Dos Santos J.N., Ribeiro D.A. (2023). Are Cytomorphogenetic Events Correlated with Oral Mucosal Lesions Induced by Crack Cocaine Use? A Systematic Review. Pathophysiology.

[18] Gohil H., Miskovic M., Buxton J.A., Holland S.P., Strike C. (2022). Smoke Gets in the Eye: A systematic review of case reports of ocular complications of crack cocaine use. Drug Alcohol. Rev..

[19] Yujra V.Q., Moretti E.G., Claudio S.R., Silva M.J., Oliveira F.d., Oshima C.T., Ribeiro D.A. (2016). Genotoxicity and mutagenicity induced by acute crack cocaine exposure in mice. Drug Chem. Toxicol..

[20] De Freitas T.A., Palazzo R.P., de Andrade F.M., Reichert C.L., Pechansky F., Kessler F., de Farias C.B., de Andrade G.G., Leistner-Segal S., Maluf S.W. (2014). Genomic instability in human lymphocytes from male users of crack cocaine. Int. J. Environ. Res. Public Health.

[21] Vijayalaxmi, Cao Y., Scarfi M.R. (2014). Adaptive response in mammalian cells exposed to non-ionizing radiofrequency fields: A review and gaps in knowledge. Mutat. Res. Mol. Mech. Mutagen..

[22] Bozorgi S.S., Proctor G.B., Carpenter G.H. (2014). Rapamycin delays salivary gland atrophy following ductal ligation. Cell Death Dis..

[23] Krishnan M., Tennavan A., Saraswathy S., Sekhri T., Singh A.K., Nair V. (2017). Acute Radiation-Induced Changes in Sprague-Dawley Rat Submandibular Glands: A Histomorphometric Analysis. World J. Oncol..

[24] Lomonosova E., Chinnadurai G. (2008). BH3-only proteins in apoptosis and beyond: An overview. Oncogene.

[25] Farhangkhoee H., Cross K.M., Koljonen V., Ghazarian D., Fish J.S. (2012). Evaluation of Ki-67 as a histological index of burn damage in a swine model. J. Burn Care Res..

[26] Lazzerini Denchi E., Attwooll C., Pasini D., Helin K. (2005). Deregulated E2F activity induces hyperplasia and senescence-like features in the mouse pituitary gland. Mol. Cell Biol..

